# Carbamazepine and Hematological Malignancies

**DOI:** 10.4274/Tjh.2013.0154

**Published:** 2013-12-05

**Authors:** İrfan Yavaşoğlu, Gökhan Sargın, Aslı Demirbulat

**Affiliations:** 1 Adnan Menderes University Medical Faculty, Division of Hematology, Aydın, Turkey; 2 Adnan Menderes University Medical Faculty, Department of Internal Medicine, Aydın, Turkey

**Keywords:** Carbamazepine, Hematological malignancies, Multiple myeloma

## TO THE EDITOR

The letter entitled “Carbamazepine and Multiple Myeloma: Possible Interaction”, written by Günaldı et al. [1] and published in one of the recent issues of your journal, was quite interesting. We would like to emphasize some points about that letter. 

In the reported case, the patient’s serum globulin level was 5.44 g/dL, and serum protein electrophoresis showed that the M-peak was closest to the beta-fraction. Thus, it becomes important to know the level of light chains and IgA as heavy chains. The serum protein electrophoresis should also be evaluated for biclonal gammopathy due to its pattern shown in Figure 1. Interleukin-6 (IL-6) is the main growth factor for multiple myeloma (MM) and studies of epileptic patients showed elevated levels of IL-6 after carbamazepine therapy [2]. We have no information about the level of IL-6 in the case reported by Günaldı et al.

The specific abnormalities such as t(4;14), t(14;16), and deletion (del) 17p detected by fluorescence in situ hybridization (FISH) analysis were reported as high-risk MM [3]. Tricot et al. [4] reported that partial or complete deletions of chromosome 13 were associated with poor prognosis in MM. Chromosome 13 deletions detected only by FISH independently in the absence of other abnormalities do not carry significantly higher risk, whereas t(11;14) does not predict superior outcome [5]. It is interesting to see cytogenetic abnormalities del17p-t(4;14)-del13 and t(11;14) in same patient. However, the consensus is that the data are not yet adequate to suggest routine use of these FISH markers to predict prognosis [5].

The number of people who had suffered from side effects due to carbamazepine was reported as 14,705. Ten of them (0.07%) had MM [6]. Drug-related (although rarely from carbamazepine) hypogammaglobulinemia is a well-known condition. 9-Acridine carboxaldehyde is one of the carbamazepine metabolites generating activated neutrophils, and presumably monocytes. This metabolite increases lymphocyte proliferations at lower concentrations and decreases them at higher concentrations. The relationship between hypogammaglobulinemia and carbamazepine is thus clearer [7]. There have also been reports of agranulocytosis, leucopenia, pure erythrocyte aplasia, and thrombocytopenia after carbamazepine [8]. Additionally, phenytoin is considered a possible carcinogenic for humans and was reported as a carcinogenic drug in animals [9].

A diagnosis of epilepsy in the same year as a cancer diagnosis carried an increased risk for leukemia, pancreatic cancer, non-Hodgkin’s lymphoma, acute myeloid leukemia, and Hodgkin’s disease, but not for chronic lymphatic leukemia or MM. The issue of cancer incidence in people with epilepsy remains an open question [9,10]. In conclusion, the answer is still unknown as to whether the drugs for epilepsy or epilepsy itself causes MM. 
